# A Manual of Surgery

**Published:** 1900-03

**Authors:** 


**A Manual of Surgery.** By CHARLES STONHAM. Three volumes. Pp. xiii., 343; xv., 383; xxi., 725. London: Macmillan and Co., Limited. 1899.

It is a common complaint amongst students that the larger text-books of surgery are too voluminous, while on the other hand the teacher or examiner often finds that the student has solely depended for his knowledge on the scanty and cramped information in the smaller (but popular) works, which are little more than "cram-books." Assuming for the moment that justice will be meted out to the contending parties by a little mutual concession in the adoption of a middle course, we may say that the present manual—one of the publishers' new series—can be regarded as a judicious compromise.

The first volume treats of general surgery. Inflammation and its associations, the infective diseases, and tumours are well described. In the section dealing with deformities, however, there is a tendency to be brief: the account of scoliosis is very meagre and there is no mention of coxa vara. Volume ii. deals with injuries. In fractures of the shaft of the femur, Liston's long splint is regarded as most convenient, instead of the much less irksome treatment by means of a weight-extension over the end of the bed. The section dealing with spinal injuries requires some revision. It is desirable to point out the cases which are unsuitable for laminectomy in fracture-dislocations of the spine, especially as we are told on p. 278 that in complete division of the cord reflex actions return in a day or two and are exaggerated. The effect of diastasis of the spinal column with a complete crush of the cord is just the converse of this. Volume iii. treats of regional surgery. "Simple epiphysitis" is treated in four lines, and the treatment of appendicitis is very brief.

A feature of the book which is most useful is an index to the whole work in each volume. The illustrations are numerous, but some of them (such as fig. 35) are lacking in artistic qualities. Regarded as a whole, the book is sound in its teaching, and the author has well utilised the space at his command. We recommend it as a step in the right direction, but still hope that students and practitioners will recognise the unwisdom of thinking that the mastery of a few dogmatically stated facts is the best means of acquiring true knowledge.

